# The neuroimmune axis in cardiovascular disease: from immune activation to inflammatory resolution

**DOI:** 10.3389/fimmu.2026.1930929

**Published:** 2026-09-24

**Authors:** Kailei Zhang, Zhichao Liu, Yancui Zhang, Yang Yang, Zexin Ding, Sitong Meng, Limin Qin, Luchang Yin, Jinhong Chen

**Affiliations:** 1 School of Rehabilitation Medicine, Shandong Second Medical University, Weifang, Shandong, China; 2 Department of Cardiovascular, Affiliated Hospital of Shandong Second Medical University, Weifang, Shandong, China

**Keywords:** autonomic nervous system, cardiovascular diseases, immunopharmacology, inflammatory resolution, neuroimmune axis, sterile inflammation

## Abstract

Cardiovascular diseases (CVDs) are driven not only by hemodynamic and metabolic stress but also by persistent sterile inflammation and defective resolution. Neuroimmune regulation has emerged as an important focus in cardiovascular research. In this review, we synthesize current evidence on the neuroimmune axis in CVDs and highlight how dysregulated autonomic immune communication promotes persistent inflammation, defective resolution, and adverse cardiovascular remodeling. We further emphasize that the neuroimmune axis may influence not only inflammatory activation but also the transition toward resolution and tissue repair, with its effects likely depending on disease stage and inflammatory context. Targeting neuroimmune pathways may help restore the temporal control of inflammation and promote cardiovascular resolution.

## 1 Introduction

Cardiovascular diseases (CVDs) remain the leading cause of mortality worldwide and continue to impose a rising health burden (1). This burden cannot be explained solely by hemodynamic load, dyslipidemia or metabolic injury (2). A central conceptual advance in cardiovascular biology is that sterile inflammation is not an accompanying feature of CVDs but a causal and therapeutically tractable process (3, 4).

Inflammation resolution is an active and programmed process, not a passive decline in inflammatory intensity (5). Specialized pro-resolving mediators (SPMs), efferocytosis, reparative macrophage polarization, regulatory T cell activity and tissue repair programs terminate acute inflammation while preserving host defense (6). Recent evidence indicates that inflammatory activation and resolution programs can begin in overlapping time windows after cardiovascular injury. This temporal overlap makes the neuroimmune axis especially important, because autonomic and immune signals may determine whether early inflammation is converted into repair or prolonged into chronic remodeling (7, 8).

This review aims to provide an overview of the neuroimmune axis in CVDs, focusing on how autonomic immune communication shapes inflammation, resolution, and cardiovascular remodeling. In particular, this review elaborates on neuroimmune anatomy, immune organ regulation, cellular crosstalk, disease specific activation, and therapeutic opportunities for restoring inflammatory timing and promoting tissue repair.

## 2 Literature search strategy

Relevant literature was identified through PubMed and Web of Science using terms related to neuroimmune regulation, cardiovascular disease, autonomic function, inflammation, and major cardiovascular conditions. English language studies published through June 2026 were considered, with priority given to original mechanistic studies, recent reviews, and clinically relevant investigations. Additional studies were identified from reference lists of key articles. As this was a narrative review, studies were selected based on their relevance to the topics discussed rather than by a predefined systematic review protocol.

## 3 Anatomical and physiological basis of the neuroimmune cardiovascular axis

### 3.1 Bidirectional communication within the neurocardiac axis

The neurocardiac axis is organized as a closed loop control system. Cardiovascular tissues generate mechanical, metabolic and inflammatory signals that are sensed by afferent nerves, integrated in brainstem and hypothalamic nuclei, returned through autonomic efferents and refined by intrinsic cardiac circuits (9). This organization provides the anatomical logic through which cardiovascular injury can be converted into coordinated immune and autonomic responses. This distinction may be useful for considering how dysregulation at different steps could contribute to pathological inflammation. Excessive afferent signaling may exaggerate central alarm responses, central autonomic nuclei may maintain sympathetic outflow despite resolution of the initiating insult, peripheral nerves may overdrive immune organs and intrinsic cardiac circuits may amplify local inflammatory reflexes (10).

#### 3.1.1 Afferent sensing of cardiovascular stress

The heart and major vessels contain sensory receptors that transmit cardiovascular information to the nucleus of the solitary tract (NTS). Arterial baroreceptors in the carotid sinus and aortic arch encode vessel stretch and pulsatility through glossopharyngeal and vagal afferents (11). Peripheral chemoreceptors, particularly those in the carotid body, detect changes in arterial oxygen tension, carbon dioxide tension, and pH (12). Cardiac sensory endings also monitor mechanical load, ischemic metabolites and nociceptive signals (13). Emerging neuroimmune evidence indicates that subsets of vagal sensory neurons can detect cytokine associated signals, providing an interoceptive route by which peripheral immune status reaches the brainstem. These afferent signals converge in the NTS for central processing (14) (**Figure 1**).

**Figure 1:**
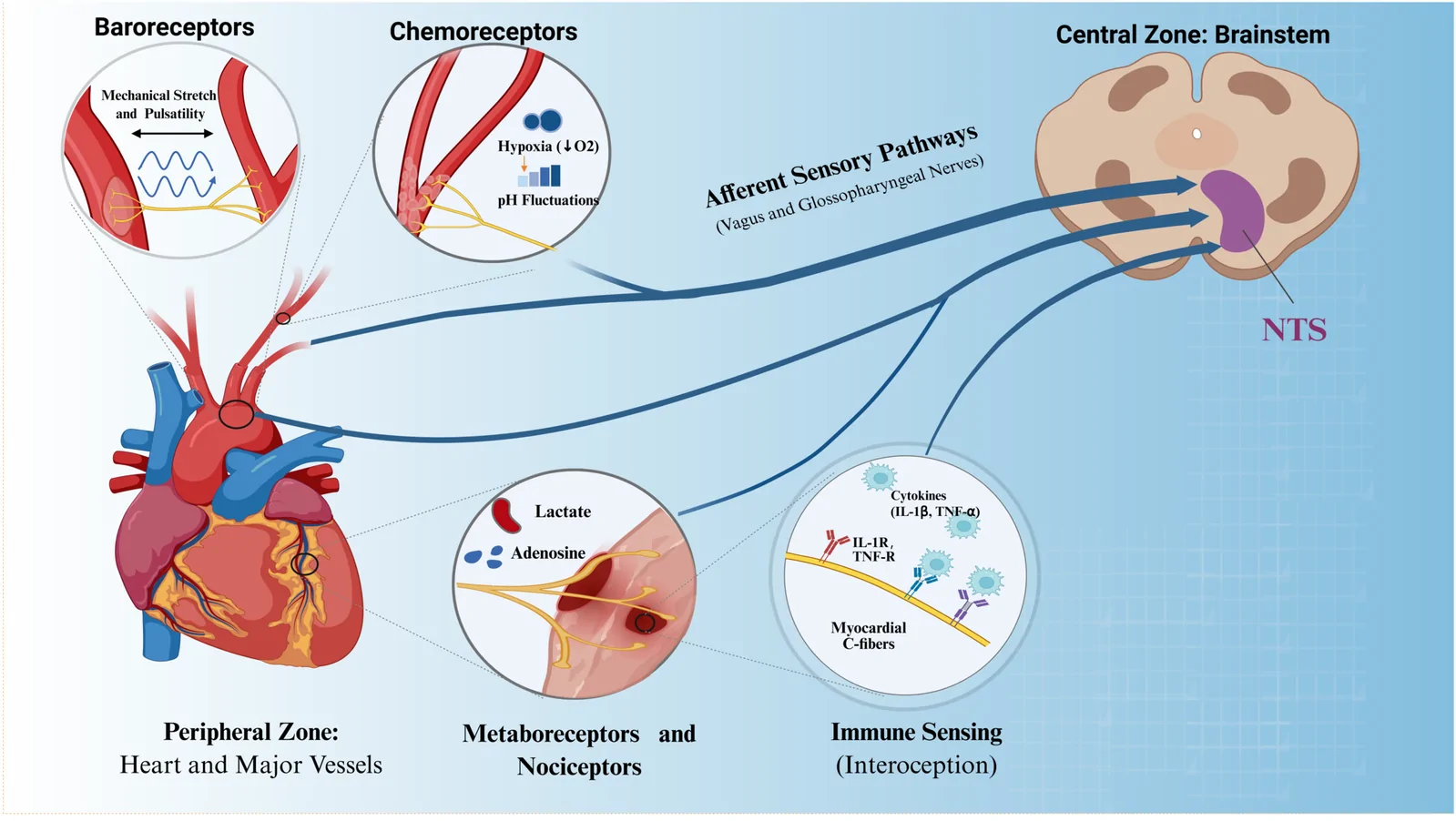
Afferent sensing of cardiovascular stress. Sensory receptors in the heart and major vessels detect mechanical stretch, vascular pulsatility, hypoxia, pH changes, ischemia related metabolites, nociceptive stimuli, and cytokine associated immune signals. These inputs are transmitted mainly through vagal and glossopharyngeal afferents and converge in the NTS, where they provide an integrated representation of cardiovascular stress, tissue injury, and peripheral immune activation. NTS, nucleus of the solitary tract; PO_2_, partial pressure of oxygen; PCO_2_, partial pressure of carbon dioxide; pH, potential of hydrogen; IL-1β, interleukin-1 beta; TNF-α, tumor necrosis factor alpha; IL-1R, interleukin-1 receptor; TNF-R, tumor necrosis factor receptor; C-fibers, unmyelinated C-type afferent fibers.

This afferent layer is also the first point at which cardiovascular inflammation becomes a neural signal. Mechanical stretch, ischemic metabolites and cytokine associated inputs are not transmitted as isolated messages but converge as integrated interoceptive information (15). The brainstem therefore receives a composite readout of hemodynamic stress, tissue injury and immune activation (16). Such convergence may be advantageous during acute injury because it coordinates cardiovascular compensation with immune defense (17). However, persistent or repeated afferent activation can bias central networks toward sustained autonomic alarm and create the upstream condition for chronic neuroimmune inflammation (8, 18).

#### 3.1.2 Central integration and reflex control

Afferent cardiovascular signals transmitted from peripheral sensory receptors must first be processed within central autonomic nuclei. The NTS serves as the primary integration center, receiving baroreceptor, chemoreceptor, metabolic and inflammatory inputs and coordinating reflexive autonomic responses through the central autonomic network (19, 20). NTS projections engage downstream brainstem nuclei, including the caudal and rostral ventrolateral medulla, which regulate sympathetic outflow through the intermediolateral column of the spinal cord (21). In parallel, interactions with the dorsal motor nucleus of the vagus and nucleus ambiguus modulate parasympathetic efferent activity controlling heart rate and atrioventricular conduction (22). Higher order regions such as the hypothalamic paraventricular nucleus integrate autonomic, endocrine and inflammatory cues, enabling state dependent cardiovascular regulation (23).

Central integration is not a passive relay. Brainstem and hypothalamic nuclei compare peripheral cardiovascular inputs with internal state variables such as stress, endocrine tone, metabolic demand and inflammatory status (24). As a result, the same peripheral stimulus can generate different autonomic outputs depending on disease stage and systemic context (24). This feature is especially relevant to chronic CVDs, in which central neuroinflammatory plasticity and altered hypothalamic signaling may maintain sympathetic dominance even after the initiating peripheral injury has begun to resolve (25, 26) (**Figure 2**).

**Figure 2:**
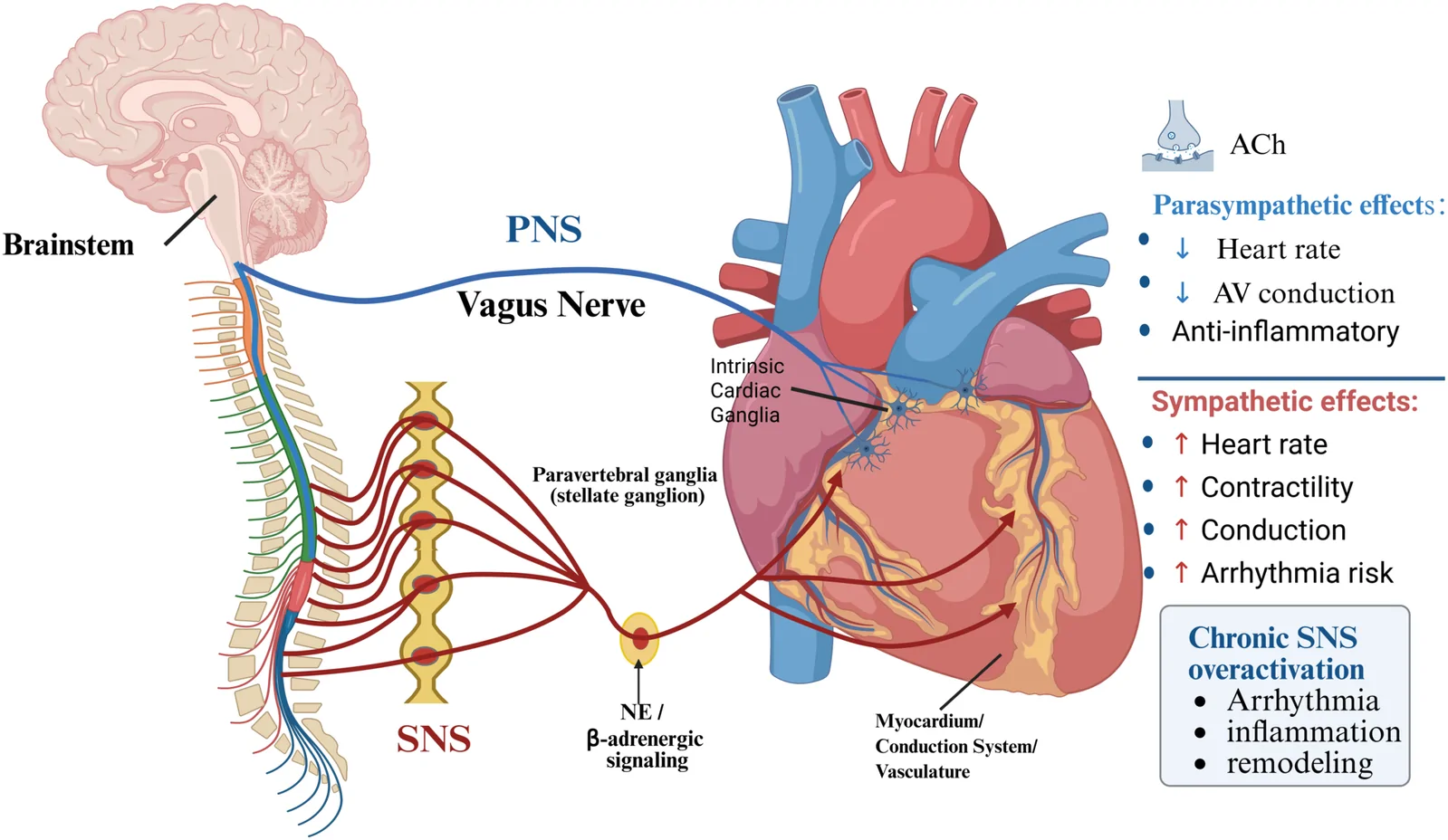
Central integration and reflex control of cardiovascular autonomic output. Cardiovascular afferent signals are centrally integrated within brainstem autonomic nuclei, particularly the nucleus of the solitary tract, and are translated into coordinated parasympathetic and sympathetic efferent responses. Vagal parasympathetic pathways regulate heart rate, atrioventricular conduction, and anti-inflammatory signaling, whereas sympathetic pathways arising through spinal and paravertebral ganglia increase heart rate, myocardial contractility, conduction, and arrhythmogenic risk via NE and β-adrenergic signaling. Sustained sympathetic nervous system overactivation may further contribute to arrhythmia, inflammation, and adverse cardiac remodeling. ACh, acetylcholine; AV, atrioventricular; NE, norepinephrine; PNS, parasympathetic nervous system; SNS, sympathetic nervous system.

#### 3.1.3 Descending autonomic output

Integrated autonomic commands are transmitted through sympathetic and parasympathetic efferent arms. Sympathetic preganglionic neurons project to paravertebral and stellate ganglia, where postganglionic fibers release NE to regulate myocardial contractility, vascular tone, conduction and immune cell activity (27). Parasympathetic output through vagal pathways releases acetylcholine in intrinsic cardiac ganglia to slow heart rate and atrioventricular conduction, and can engage cholinergic anti-inflammatory mechanisms that restrain excessive cytokine production in selected peripheral immune contexts (28). Disease progression is favored when autonomic balance shifts toward sustained sympathetic overactivity and parasympathetic withdrawal (29).

#### 3.1.4 Intrinsic cardiac nervous system and local integration

Beyond central autonomic output, the heart contains an intrinsic cardiac nervous system composed of ganglionated plexi embedded within epicardial fat pads and atrial regions (30). These local circuits integrate sympathetic and vagal inputs, shape regional excitability and coordinate contractile responses (31). The intrinsic cardiac nervous system therefore acts as a peripheral processing layer that can translate systemic stress and local inflammatory cues into chamber specific autonomic and immune effects (32).

### 3.2 Structural basis of the neuroimmune interface in lymphoid organs

Autonomic nerves also innervate immune organs, creating an anatomical bridge through which cardiovascular stress can be translated into systemic immune remodeling. This bridge is crucial because many inflammatory cells that infiltrate vessels and myocardium originate from or are activated in lymphoid organs rather than within cardiovascular tissue itself (33).

#### 3.2.1 Sympathetic innervation of immune organs

Postganglionic sympathetic fibers innervate primary and secondary lymphoid organs, including bone marrow, thymus, spleen and lymph nodes (34). This innervation positions NE signaling near hematopoietic and immune cell niches, allowing autonomic output to influence hematopoietic stem and progenitor cell (HSPC) retention and mobilization, antigen dependent lymphocyte activation and leukocyte trafficking (35). The relevance of this architecture is functional, changes in sympathetic tone can alter the quantity and phenotype of immune cells delivered to cardiovascular lesions (36).

#### 3.2.2 The vagal splenic and cholinergic anti-inflammatory interface

Parasympathetic regulation of systemic inflammation is mediated largely through the cholinergic anti-inflammatory pathway. Current models support an indirect vagal splenic relay involving autonomic ganglia, the splenic nerve and acetylcholine producing immune cells rather than simple direct vagal innervation of the spleen (37). Through α7 nicotinic acetylcholine receptor (α7nAChR) signaling, this pathway can suppress excessive cytokine release and complement sympathetic regulation of immune cell mobilization (38).

Importantly, immune organ innervation makes the neuroimmune axis systemic rather than purely local (37). A small change in autonomic output can alter hematopoietic production, splenic release, lymphocyte trafficking and tissue infiltration across multiple vascular beds (39). This organization may help link focal cardiovascular injury to inflammation at distant vascular sites and may also explain how chronic stress primes immune cells before they enter the heart or arterial wall. The anatomical scaffold therefore creates both risk and opportunity, because it can distribute inflammation widely, but it can also be targeted upstream to normalize immune supply before tissue damage becomes irreversible (40).

## 4 Mechanisms of neuroimmune regulation in cardiovascular inflammation

Through its regulation of cardiovascular inflammation and resolution, the neuroimmune system links systemic leukocyte supply, local injury repair responses, and reciprocal neural remodeling in CVDs.

### 4.1 Neuroimmune organ axes in cardiovascular inflammation and resolution

The neuroimmune organ axis is the structural framework through which neural circuits regulate systemic immune activity. Autonomic signals alter immune cell production, mobilization and activation in bone marrow, spleen, lymph nodes, adventitial tertiary lymphoid organs (ATLOs) and thymus (33, 41). In return, immune derived mediators remodel neural excitability and autonomic output, forming loops that can either contain inflammation or amplify it (42, 43).

An organ level view also clarifies why resolution failure can originate outside the target tissue. A plaque, infarct or hypertensive vessel may fail to resolve not only because local macrophages are dysfunctional, but also because bone marrow and splenic reservoirs continue to deliver inflammatory cells (44).

In this sense, cardiovascular resolution depends on both local clearance mechanisms and distal immune organ restraint (45). The key question is therefore not whether a given organ is involved in one specific disease, but how its neural regulation changes the quality, timing and destination of immune cell output (**Figure 3**).

**Figure 3:**
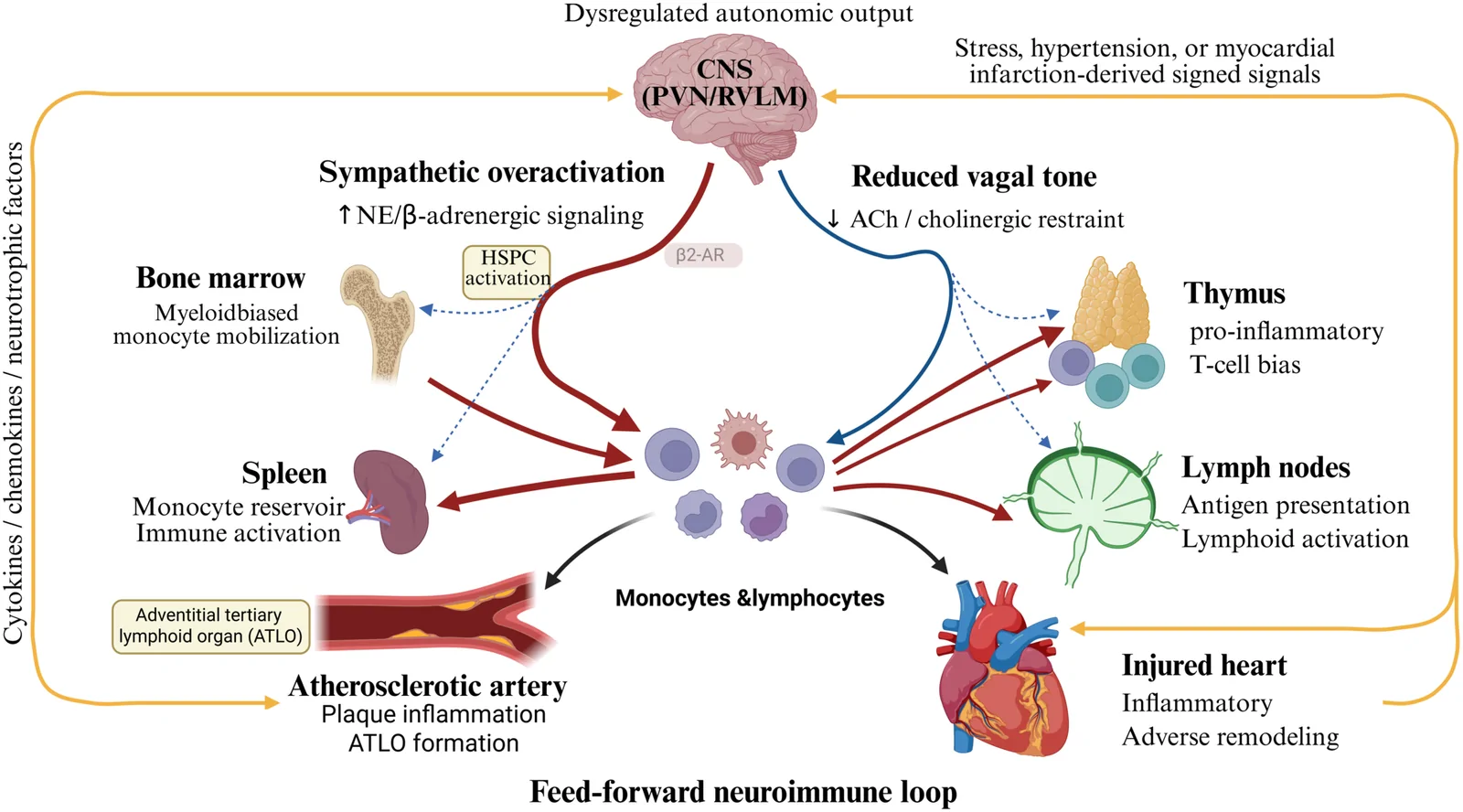
Neuroimmune organ axes in cardiovascular inflammation. Dysregulated autonomic output during cardiovascular stress enhances sympathetic NE/β-adrenergic signaling and reduces vagal cholinergic restraint. This imbalance promotes bone marrow HSPC activation, splenic monocyte mobilization, lymphoid immune activation, and pro-inflammatory T cell bias, thereby increasing circulating monocytes and lymphocytes that aggravate vascular and myocardial inflammation. Cytokines, chemokines, and neurotrophic signals from injured cardiovascular tissues further feedback to central autonomic circuits, forming a feedforward neuroimmune loop that sustains inflammation and adverse remodeling. ACh, acetylcholine; ATLO, adventitial tertiary lymphoid organ; β2-AR, β2-adrenergic receptor; CNS, central nervous system; HSPC, hematopoietic stem and progenitor cell; NE, norepinephrine; PVN, paraventricular nucleus; RVLM, rostral ventrolateral medulla.

#### 4.1.1 Bone marrow

Bone marrow is the principal source of circulating innate immune cells and a central neuroimmune hub in cardiovascular inflammation (46–48). In homeostasis, autonomic and neuroendocrine inputs maintain HSPC quiescence, circadian trafficking and niche retention, thereby limiting unnecessary myeloid output and preventing sterile inflammatory infiltration of vessels and myocardium (49).

During cardiovascular stress, danger associated signals and central autonomic activation can enhance sympathetic input to the bone marrow, thereby promoting HSPC activation and inflammatory myeloid output (50). NE signaling reshapes the niche by promoting HSPC proliferation, weakening retention pathways such as CXCL12 and biasing differentiation toward inflammatory myeloid lineages. This is best interpreted as a shared marrow stress program rather than a mechanism unique to one disease (10, 50). Experimental evidence supports this sequence. Chronic stress expands inflammatory leukocyte production through HSPC activation, while cardiovascular injury can couple sympathetic activation to myeloid mobilization and trained immunity like epigenetic remodeling in monocytes and their progenitors (51, 52).

Thus, inflammatory myeloid cell supply to cardiovascular lesions may be sustained by sympathetic and injury induced activation of bone marrow hematopoiesis, thereby delaying the transition from debris clearance to tissue repair (51, 53). Bone marrow may therefore be regarded as an adaptive neuroimmune organ whose output is recalibrated by sympathetic and epigenetic cues during cardiovascular injury (46).

#### 4.1.2 Spleen

The spleen is a rapidly deployable immune reservoir that links systemic inflammatory stress to cardiovascular tissue inflammation (54). Its key relevance lies in the capacity to store monocytes, coordinate antigen dependent immune activation and release inflammatory cells into the circulation when neural and humoral stress signals increase (55).

Under basal conditions, sympathetic and cholinergic inputs help tune splenic cytokine production and immune cell trafficking (56). During inflammatory stress, sympathetic predominance favors monocyte mobilization and pro-inflammatory cytokine output, whereas vagal cholinergic signaling constrains tumor necrosis factor alpha and IL-1β release through α7nAChR (57). Thus, the spleen functions as a dynamic checkpoint that can either amplify systemic inflammation or support its containment.

In mouse models, splenic Ly6C^hi^ monocytes rapidly decline after infarction while their accumulation increases in injured myocardium, indicating release from the splenic reservoir and recruitment to the heart (58). These findings support a spleen sympathetic heart axis that shapes the early magnitude of post injury inflammation. Insufficient cholinergic restraint may allow splenic immune output to prolong systemic inflammation and delay resolution.

#### 4.1.3 Lymph nodes, ATLOs and thymus

Beyond bone marrow and spleen, lymph nodes, ATLOs and thymus provide distinct neuroimmune relays. Their relevance lies in antigen specific priming, lesion directed immune organization and long-term T cell repertoire maintenance rather than bulk leukocyte supply (59, 60).

Lymph nodes regulate adaptive immune trafficking and antigen driven activation (59). Experimental evidence indicates that β2 adrenergic signaling controls lymphocyte egress from lymph nodes, positioning sympathetic tone as a regulator of when immune cells leave lymphoid tissues and reach inflamed cardiovascular sites (61). In chronic vascular inflammation, this mechanism may sustain antigen dependent T cell and B cell output, whereas cholinergic restraint can limit cytokine programs and support immune containment (62, 63).

ATLOs are specialized lymphoid structures that arise in the arterial adventitia during advanced atherosclerosis (64). They contain organized B cell and T cell zones, high endothelial venules and antigen presenting niches (65). Recent studies suggest that ATLOs acquire sympathetic and sensory innervation during plaque evolution and that resident immune cells produce neurotrophic cues that remodel local nerve fibers (66). ATLOs have been proposed to function as local neuroimmune interfaces that coordinate plaque associated immune activation and may contribute to impaired inflammatory resolution (41). However, direct evidence for bidirectional functional interactions between ATLOs and local nerves remains limited, and their causal contribution to plaque progression and impaired inflammatory resolution has yet to be established.

The thymus contributes through T cell education, immune aging and tolerance. Autonomic inputs and catecholamine production by thymic epithelial cells participate in intrathymic T cell maturation (60, 67). Experimental studies have shown that early life thymectomy reduces naive T cell output while promoting the accumulation of senescent pro-inflammatory T cells, changes that are accompanied by arterial stiffening and endothelial dysfunction (68). Thymic dysfunction may therefore skew T cell output toward pro-inflammatory phenotypes and weaken tolerance mechanisms that normally restrain chronic cardiovascular inflammation (68).

### 4.2 Cellular neuroimmune crosstalk in cardiovascular injury and resolution

Inflammation resolution at the cellular level requires more than a decline in leukocyte numbers (69). It is driven by coordinated shifts in cell function and phenotype, some of which are regulated by neural mediators (70). Thus, cellular neuroimmune crosstalk represents an important mechanism by which persistent tissue damage may be limited and reparative remodeling may be promoted (**Figure 4**).

**Figure 4:**
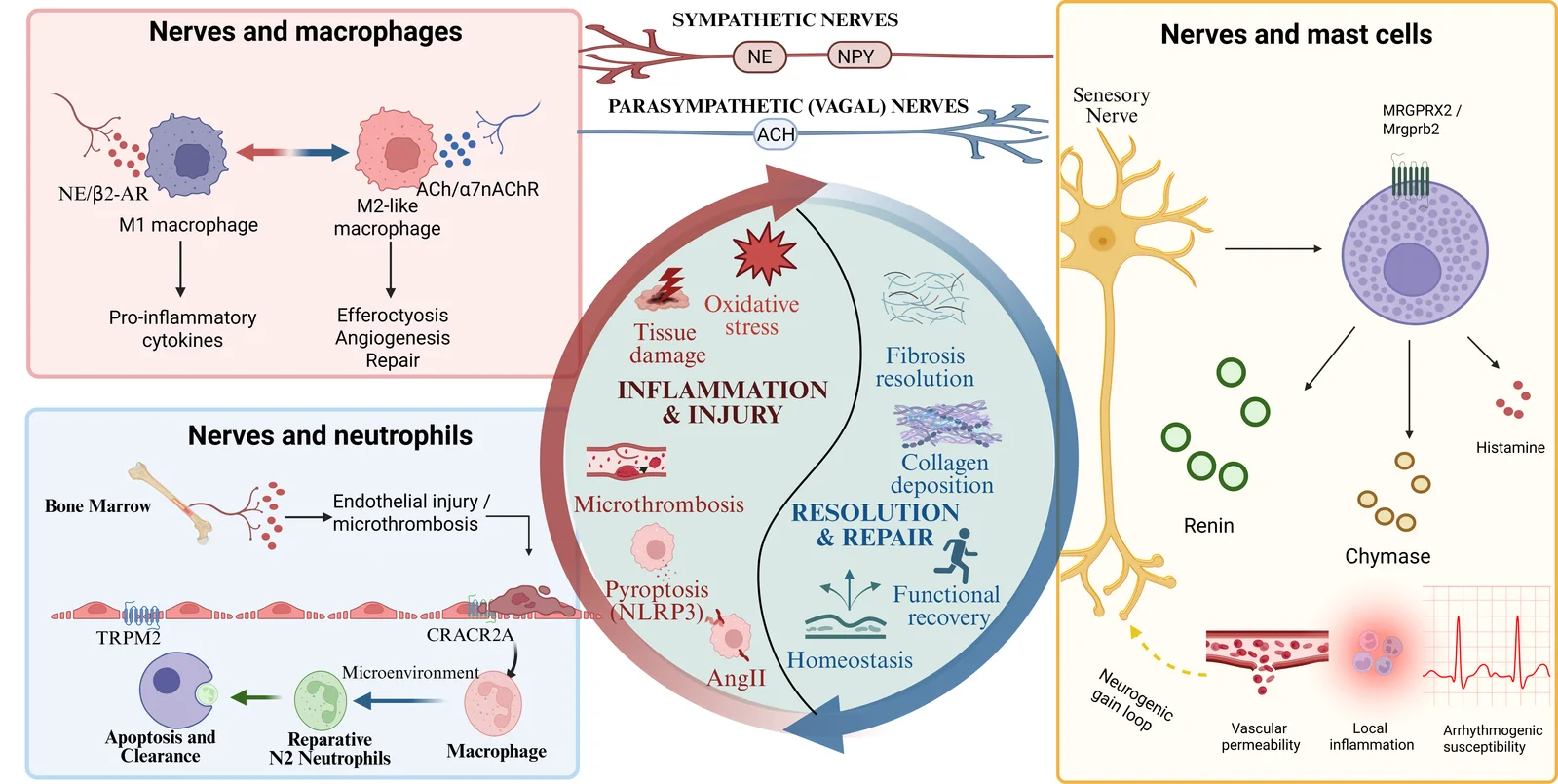
Cellular neuroimmune crosstalk in cardiovascular inflammation and repair. Neural signals regulate immune cell responses within injured cardiovascular tissues. Sympathetic mediators, such as NE and neuropeptide Y, promote inflammatory activation, leukocyte recruitment, oxidative stress, microthrombosis, and tissue damage, whereas vagal acetylcholine signaling limits excessive inflammation and facilitates resolution. These inputs modulate macrophages, neutrophils, mast cells, thereby influencing the transition from persistent inflammation to fibrosis resolution, tissue repair, and functional recovery. ACh, acetylcholine; Ang II, angiotensin II; NE, norepinephrine; NPY, neuropeptide Y.

#### 4.2.1 Nerves and macrophages

Macrophages are central to cardiovascular resolution because they link inflammatory sensing with efferocytosis, matrix remodeling, and tissue repair (71). Macrophage recruitment, cytokine production, polarization, and clearance capacity may be shaped by neural inputs, whereas autonomic mediators such as NE and acetylcholine further regulate macrophage inflammatory and reparative programs (69). Sympathetic catecholamine signaling through β2 adrenergic receptors can promote pro-inflammatory cytokine programs in macrophages in selected contexts, whereas cholinergic signaling through α7nAChR suppresses macrophage TNF-α production and restrains excessive inflammatory amplification (72, 73). These opposing inputs position macrophages along an inflammatory to reparative continuum.

After myocardial injury, nerve terminals and infiltrating macrophages accumulate near the infarct border zone, enabling rapid neurogenic conditioning of macrophage responses (74). Sympathetic hyperactivation favors persistence of inflammatory macrophages and adverse remodeling, while parasympathetic or cholinergic signaling supports M2 like reparative polarization, efferocytosis, matrix organization and angiogenesis (75, 76). Neural inputs therefore regulate both the intensity of macrophage inflammation and the timing of resolution.

This temporal context is important for macrophage targeted therapy. Broad inhibition of macrophage activation during the early phase may impair necrotic debris clearance, whereas insufficient efferocytosis and reparative polarization during later stages may prolong inflammation and fibrosis (77).

#### 4.2.2 Nerves and neutrophils

Neutrophils are early responders in sterile cardiovascular injury, and neural inputs influence their mobilization, endothelial entry and clearance (78). Sympathetic signaling regulates these steps indirectly by altering endothelial activation and bone marrow egress (79). At the vascular interface, endothelial transient receptor potential melastatin 2 facilitates paracellular passage, whereas neutrophil calcium release activated calcium channel regulator 2A supports calcium signaling, integrin activation and vascular retention. These molecules may be viewed as downstream entry and retention mechanisms that operate within a neuroinflammatory environment (80, 81).

Once activated, neutrophils amplify tissue injury through reactive oxygen species, proteases and neutrophil extracellular traps (NETs). NETs promote endothelial damage, oxidative stress, microthrombosis and thrombo inflammatory signaling (82). In early myocardial injury models, β3 adrenergic signaling downregulates CXCL12 expression in bone marrow stromal cells, accelerating neutrophil egress into the bloodstream and increasing inflammatory delivery to injured myocardium (83, 84).

Resolution requires active termination of the neutrophil phase. Neutrophil apoptosis, macrophage mediated efferocytosis and context dependent transitions toward reparative neutrophil states help prevent persistent tissue damage (85). When clearance is defective, neutrophils are retained in injured tissue, toxic granule contents accumulate and inflammation fails to resolve efficiently (86). Thus, sympathetic inputs amplify early neutrophil supply, whereas local clearance checkpoints determine whether neutrophil driven injury is converted into repair (87).

Neural regulation of neutrophils therefore has a narrow therapeutic window. Early excessive sympathetic drive expands the neutrophil wave and increases thrombo inflammatory injury, but complete prevention of neutrophil entry may compromise pathogen defense and debris processing (88).

#### 4.2.3 Nerves and mast cells

Mast cells couple sensory nerves to rapid vascular inflammatory amplification. Substance P released from sensory fibers activates mast cells predominantly via MRGPRX2/Mrgprb2, triggering degranulation with histamine and chymase release and thereby increasing vascular and endothelial permeability (89, 90). Neuropeptide Y may modulate mast cell and immune cell output in a receptor dependent manner, whereas sensory neuropeptides such as substance P provide a more established mechanism by which neuropeptide signaling lowers the threshold for mast cell driven leukocyte entry and local inflammation (91).

Mast cell mediators also feedback to nerves and create a neurogenic gain loop (92). In ischemia reperfusion and plaque inflammation, sensory neuropeptide mast cell crosstalk and mast cell mediators, including renin and chymase related local renin angiotensin signaling, can amplify vascular inflammation, myocardial injury, and arrhythmogenic susceptibility (93). Rather than acting through isolated neuropeptide pathways, communication between nerves and mast cells appears to form a reciprocal signaling loop that can amplify cardiovascular inflammation (92).

During chronic inflammation, mast cells may adopt tissue and microenvironment shaped programs characterized less by stereotyped explosive degranulation and more by selective mediator release, cellular crosstalk, and modulatory functions (94). Microenvironment dependent changes in mast cell mediator and protease profiles may recalibrate nerve mast cell coupling, potentially shifting bidirectional neuroimmune signaling from persistent amplification toward more context dependent modulation (95, 96).

### 4.3 Immune to neural feedback regulation

Neuroimmune communication is reciprocal rather than unidirectional. Immune cells and lymphoid structures feedback to neural circuits through cytokines, chemokines, neurotrophic factors and changes in neurotransmitter metabolism (97, 98). These signals can alter neuronal excitability, nerve density, receptor sensitivity and autonomic output, creating self-reinforcing loops that sustain cardiovascular inflammation (46).

Feedback regulation is particularly relevant to chronic disease because it can preserve inflammatory memory after the initial cardiovascular stimulus has declined (99). Cytokine exposure, neurotrophic remodeling and immune driven changes in neurotransmitter metabolism may reset autonomic tone, making neural circuits more responsive to subsequent stress (100). Once this reset occurs, each new inflammatory episode is processed by a sensitized neural network, increasing the probability of recurrent immune activation. This provides a mechanistic bridge between acute injury, chronic remodeling and recurrent cardiovascular events (101).

In atherosclerosis, ATLOs provide a local vascular wall immune niche that organizes adventitial immune responses and may participate in reverse neuroimmune regulation (41). Neurotrophic cues and chemokines released by immune cells within ATLOs may contribute to sympathetic and sensory nerve remodeling in the adventitia. In turn, these remodeled nerves may influence plaque associated immune activity and sustain local inflammation or impair inflammatory resolution (102). Lymphoid structures may therefore both respond to neural input and shape local autonomic remodeling.

## 5 Disease specific neuroimmune activation and resolution failure in CVDs

Neuroimmune imbalance does not lead to a single cardiovascular phenotype. Across hypertension, atherosclerosis, myocardial infarction (MI), arrhythmia, and heart failure (HF), related neural and immune pathways are recruited at different sites and disease stages. Failed resolution therefore manifests differently, ranging from vascular remodeling and plaque instability to adverse cardiac repair, electrical vulnerability, progressive fibrosis and contractile dysfunction. The major disease-specific neural pathways, immune mechanisms, and characteristic patterns of failed inflammatory resolution are summarized in **Table 1**.

**Table 1:** Summary of representative neuroimmune pathways, immune mechanisms, and patterns of failed inflammatory resolution across major cardiovascular diseases.

Disease	Key neural pathways	Key immune mechanisms	Failed inflammatory resolution	Representative references
Hypertension	Renal afferent activation; central sympathetic overactivity	Vascular immune activation; cytokine and oxidative signaling	Reduced pro-resolving signaling; impaired efferocytosis and neutrophil clearance	(103–105)
Atherosclerosis	Sympathetic regulation of bone marrow and spleen; adventitial/ATLO signaling	Monocyte and neutrophil recruitment; lymphocyte activation; macrophage persistence	Impaired efferocytosis; imbalance of pro-inflammatory and pro-resolving mediators	(106–109)
Myocardial infarction	Afferent injury sensing; central autonomic activation; sympathetic output to immune organs	Leukocyte mobilization; macrophage clearance and reparative polarization	Failure of the transition from inflammation to repair	(110–113)
Arrhythmia	Stellate ganglion inflammation; sympathetic sensitization and neural sprouting	T cell and macrophage infiltration; local inflammatory signaling	Persistent ganglionic inflammation; sympathetic immune feed forward amplification	(114–117)
Heart failure	Sympathetic overactivity; reduced vagal restraint; immune to neural feedback	Inflammatory myelopoiesis; leukocyte recruitment; macrophage activation	Inadequate efferocytosis; persistent inflammation and fibrosis	(118–120)

ATLO, adventitial tertiary lymphoid organ; HF, heart failure; MI, myocardial infarction.

Representative references indicate selected studies supporting the disease specific mechanisms summarized in the table.

### 5.1 Hypertension

In hypertension, abnormal central autonomic integration and enhanced renal afferent input sustain sympathetic activation. This neurogenic response promotes immune cell activation and vascular infiltration, leading to endothelial dysfunction and increased vascular contractility (103). Under these conditions, vascular and splenic immune cells acquire a pro-inflammatory phenotype. Their cytokine and oxidative outputs further amplify vascular inflammation, impair endothelial function, and promote vascular remodeling (121). These mediators promote vasoconstriction, sodium retention and end organ injury (122). At the same time, regulatory T cells and reparative macrophage programs are reduced or functionally insufficient, weakening endogenous anti-inflammatory control (123).

Resolution fails because persistent angiotensin II and neurogenic inflammatory signaling impair mediator generation and blunt protective renin angiotensin signaling (105, 124). In this setting, impaired pro-resolving mediator signaling may weaken efferocytosis and neutrophil clearance, while persistent neuroinflammation helps maintain sympathetic output (125). The clinical consequence is sustained blood pressure elevation with vascular stiffening, endothelial dysfunction and progressive target organ injury (126). Within this framework, hypertension is not simply pressure elevation followed by inflammation. It is a state in which neural and immune systems cooperate to maintain vascular tone, renal sodium handling and endothelial activation beyond normal regulatory limits (104).

### 5.2 Atherosclerosis

Atherosclerosis is a chronic inflammatory disease in which neuroimmune dysregulation and impaired resolution jointly drive plaque progression and instability (106). Neural regulation may involve sympathetic remodeling of immune reservoirs, vascular adventitial innervation, and ATLO associated neuroimmune interfaces, which may collectively contribute to monocyte recruitment, antigen driven lymphocyte activation, and local cytokine production within the arterial wall (46, 107). Inflammatory amplification is sustained when bone marrow and splenic outputs deliver monocytes and neutrophils to plaques, while local adventitial neuroimmune circuits maintain immune activation (106). Failed inflammatory resolution involves inadequate efferocytosis and the persistence of inflammatory macrophages and neutrophils. It is also accompanied by an imbalance between pro-inflammatory mediators and specialized pro-resolving lipid mediators, including resolvins and lipoxins (108, 127).

From a resolution centered viewpoint, plaque progression represents a spatial failure of containment. Early inflammatory recruitment may help clear modified lipoproteins and dead cells, but persistent neural and immune organs input overwhelms local efferocytosis and stabilizing matrix repair (107, 128). ATLO associated nerve remodeling may further localize this imbalance within the arterial adventitia and contribute to sustained immune activation adjacent to the plaque (109).

### 5.3 Myocardial infarction

MI is initiated by acute ischemic injury, whereas subsequent tissue damage and repair are strongly shaped by neuroimmune activation (76). Through afferent sensing, central autonomic integration, and sympathetic output to the bone marrow and spleen, inflammatory leukocyte mobilization is enhanced, post-infarction inflammation is amplified, and secondary myocardial injury is promoted (110). Early inflammation is required for necrotic debris clearance, but this response must subsequently shift toward macrophage mediated efferocytosis and reparative remodeling. Failure of this transition leads to prolonged inflammation, excessive collagen deposition and adverse ventricular remodeling (111, 129). Resolution after MI is shaped by coordinated local and central neuroimmune mechanisms (99). At the tissue level, local guidance cue signaling contributes to the resolution of postinfarction inflammation. Semaphorin 3A has been shown to facilitate inflammatory resolution after MI, supporting a role for neural guidance cues in the transition from injury to repair (112). In parallel, central autonomic inflammatory circuits involving brainstem and hypothalamic signaling may sustain sympathetic activation, leukocyte mobilization, and myocardial inflammation after infarction. Suppression of these maladaptive central circuits may attenuate myocardial inflammation and preserve cardiac function. Clinically, failure of the injury to repair transition promotes scar expansion, HF, and arrhythmogenic remodeling (130).

These findings suggest that future therapeutic strategies after MI may require temporal selectivity. During the first hours to days, excessive sympathetic activation and leukocyte mobilization may need to be restrained to limit secondary injury (131). During the reparative phase, therapeutic emphasis may shift toward macrophage efferocytosis, mediator programs that promote resolution, angiogenesis, and scar maturation. Broad suppression of inflammation without attention to timing could impair early debris clearance or provide insufficient support for later repair (113).

### 5.4 Arrhythmia

Arrhythmogenesis is increasingly recognized as a neuroimmune driven process in which inflammation within cardiac autonomic structures amplifies sympathetic excitability. The stellate ganglion is a key site of this interaction because local neuroinflammation can sensitize sympathetic neurons and increase abnormal cardiac sympathetic discharge (114). After myocardial injury or HF, inflammatory remodeling of the stellate ganglion can activate nerve growth factor tropomyosin receptor kinase A signaling, promote neural sprouting and intensify sympathetic input to the myocardium (132, 133). Similar inflammatory signatures, including T cell and macrophage infiltration, have been observed in stellate ganglia from patients with severe ventricular arrhythmias and inherited ion channel disorders (115). Conversely, local IL-10 overexpression in the stellate ganglion has been shown to suppress sympathetic nerve activity, attenuate myocardial inflammation, improve heart rate variability, and reduce the incidence of ventricular arrhythmias (116). These findings place ganglionic inflammation upstream of autonomic imbalance rather than treating it as a secondary electrophysiological detail. Resolution fails when anti-inflammatory signals such as IL-10 and vagal cholinergic tone cannot suppress ganglionic inflammation (38). A feed forward loop then develops between sympathetic discharge and local immune activation, increasing susceptibility to ventricular arrhythmias, electrical storm and atrial arrhythmia recurrence (116).

These findings suggest that targeting neuroimmune mechanisms may represent a potential future strategy for arrhythmia management beyond conventional cardiomyocyte ion channel modulation. In susceptible patients, inflammation within autonomic ganglia may amplify sympathetic input to vulnerable myocardium and contribute to recurrence (134). Anti-inflammatory strategies targeting the stellate ganglion may therefore complement antiarrhythmic drugs and ablation by limiting the neuroinflammatory substrate that sustains sympathetic overactivity (117).

### 5.5 Heart failure

HF can be understood as a state of chronic neuroimmune remodeling, in which autonomic imbalance sustains myocardial inflammation and progressively drives ventricular remodeling (118). Hemodynamic overload and myocardial injury activate cardiac afferent signaling, which is integrated by central autonomic nuclei and translated into altered sympathetic and vagal output (100). The resulting sympathetic overactivity and reduced vagal restraint may persist beyond the initiating insult, sustaining altered bone marrow output, splenic leukocyte release, and immune activity within the failing myocardium (119). Inflammation is amplified as sympathetic signaling promotes inflammatory myelopoiesis and monocyte and neutrophil recruitment to the failing heart (135). Within the failing myocardium, cytokine and chemokine gradients promote macrophage activation and leukocyte retention. Persistent oxidative stress and endothelial dysfunction then reinforce damage associated inflammatory signaling, allowing local inflammation to be sustained (136). Fibroblast derived signals and extracellular matrix remodeling further reinforce this inflammatory niche and link immune activation to progressive structural remodeling (120). Immune to neural feedback may stabilize this maladaptive state, as cytokines, chemokines, and neurotrophic factors released by activated immune and stromal cells can increase autonomic excitability and sympathetic dominance (137, 138).

Resolution is impaired when persistent inflammatory leukocyte recruitment is not matched by effective repair programs that restore myocardial homeostasis (8). In this chronic setting, the transition toward reparative phenotypes is impaired by inadequate efferocytosis and persistent inflammatory macrophage activation within the failing myocardium, thereby sustaining maladaptive ventricular remodeling (8). As fibroblast activation and matrix deposition persist, unresolved inflammation becomes embedded in ventricular remodeling, contributing to progressive fibrosis, contractile dysfunction, and electrical vulnerability (120).

## 6 Therapeutic opportunities for CVDs targeting the neuroimmune axis

Therapeutic translation should be guided by restoration of neuroimmune timing rather than indiscriminate immune suppression (139). Interventions should be aligned with disease stage, because early cardiovascular injury requires controlled inflammatory clearance, whereas chronic remodeling requires resolution and repair (140). Circuit level neuromodulation, pharmacology, and cell or material-based strategies may target complementary checkpoints in autonomic imbalance, inflammatory signaling, and immune cell programming (**Figure 5**).

**Figure 5:**
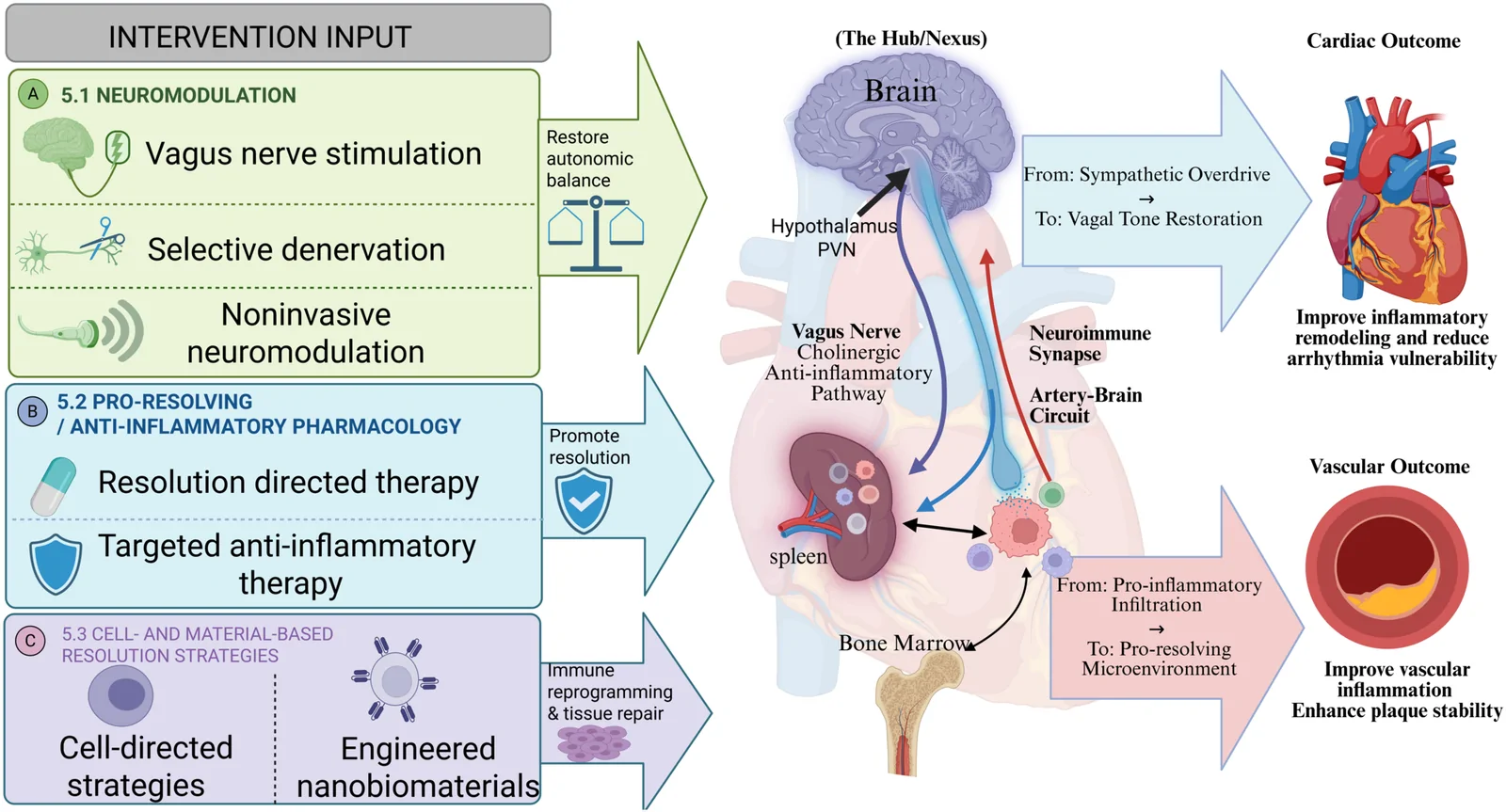
Therapeutic strategies targeting neuroimmune regulation in cardiovascular inflammation. Neuromodulation, pro-resolving and anti-inflammatory pharmacology, and cell and material-based therapies may restore autonomic balance, promote inflammatory resolution, and reprogram immune responses. These interventions act on central and peripheral neuroimmune hubs, including the brain, vagal cholinergic pathways, spleen, bone marrow, and artery brain circuits, shifting cardiovascular inflammation from sympathetic overdrive and pro-inflammatory infiltration toward vagal tone restoration, tissue repair, and plaque stabilization. Ultimately, these approaches may reduce adverse cardiac remodeling, arrhythmia vulnerability, vascular inflammation, and plaque instability. PVN, paraventricular nucleus; Th17, T helper 17 cell.

### 6.1 Neuromodulation

Vagus nerve stimulation engages the cholinergic anti-inflammatory pathway and can reduce macrophage cytokine production, splenic immune activation and vascular inflammation in experimental settings (38, 72). Selective denervation strategies, including splenic or stellate ganglion interventions, can attenuate sympathetic inflammatory drive in hypertension, myocardial injury and arrhythmia models (141). However, invasive denervation is difficult to reverse and may be followed by adaptive autonomic remodeling. Clinical translation therefore requires careful patient selection, safety monitoring, and biomarkers of on target circuit engagement (142).

Noninvasive neuromodulation is emerging as a complementary approach. Focused or transcranial ultrasound strategies have been reported to regulate central neuroinflammation, hypothalamic signaling and sympathetic output in preclinical cardiovascular injury models (143). These methods are attractive because they may target central neuroimmune hubs without surgical denervation, but standardized stimulation parameters, anatomical targeting and long-term neurovascular safety remain essential prerequisites (144).

### 6.2 Pro-resolving and anti-inflammatory pharmacology

Pharmacological strategies may help restore neuroimmune resolution once inflammatory activation has become persistent. Resolution directed therapy is designed to promote endogenous termination programs rather than indiscriminately suppress immune activity (145). SPM-based agents, including lipoxin, resolvin, protectin, and maresin analogues, as well as receptor targeted compounds, may limit neutrophil recruitment, enhance macrophage efferocytosis, promote reparative polarization, and support tissue repair (124). Their clinical development remains constrained by molecular stability, tissue delivery, receptor selectivity, therapeutic window, and pharmacodynamic biomarkers of resolution pathway activation (146).

Targeted anti-inflammatory therapy may provide a complementary approach when cytokine driven remodeling remains dominant (136). Clinical evidence has supported the therapeutic potential of inflammation modulation in cardiovascular diseases. The Canakinumab Anti-inflammatory Thrombosis Outcomes Study (CANTOS) demonstrated that selective inhibition of IL-1β signaling reduced recurrent cardiovascular events in selected patients with previous myocardial infarction and persistent inflammatory activation (147). The COLCOT and LoDoCo2 trials further showed that low-dose colchicine reduced cardiovascular events in patients after myocardial infarction and in those with chronic coronary disease, respectively (148, 149). Established cardiovascular drugs may also intersect with this framework (148). Statins may partly support resolution through effects on SPMs, whereas β blockers may reduce catecholamine driven leukocyte mobilization and inflammatory amplification during sustained sympathetic activation (139, 150). These approaches may need to be tailored to disease stage to avoid disrupting protective inflammatory responses.

### 6.3 Cell and material based resolution strategies

Cell directed approaches aim to redirect immune programs rather than eliminate immune activity (145). Strategies that enhance regulatory T cell function, restrain pathogenic Th17 responses or promote reparative macrophage polarization may restore inflammatory balance in hypertension, myocardial injury and vascular disease (151, 152). Engineered nano-biomaterials loaded with metabolic precursors illustrate how macrophage metabolism can be redirected toward bilirubin and carbon monoxide production, promoting tissue repair after myocardial injury (153). For clinical translation, biodistribution, scalability, immunogenicity and long-term safety must be systematically evaluated (151, 154).

## 7 Conclusion

Across cardiovascular diseases, neuroimmune dysregulation has been increasingly recognized as a common link between autonomic imbalance, persistent inflammation, impaired resolution, and adverse remodeling. However, the predominant mechanisms appear to vary across disease contexts. Systemic and vascular immune activation is particularly implicated in hypertension and atherosclerosis, whereas outcomes after myocardial infarction are strongly influenced by the transition from inflammatory clearance to tissue repair. In arrhythmia and heart failure, persistent autonomic inflammation and neural remodeling have been increasingly associated with electrical and structural dysfunction.

Despite substantial mechanistic advances, the strength of available evidence remains variable. Autonomic regulation of immune cell mobilization and inflammatory activity is supported by considerable experimental evidence, whereas the causal roles of several local neuroimmune circuits remain less well established, particularly in humans. Clinical translation is further limited by uncertainties regarding intervention timing, target selection, patient stratification, and reliable biomarkers of neuroimmune activity and inflammatory resolution.

Future studies should therefore incorporate anatomical context, disease stage, and resolution status into experimental and clinical design. Longitudinal human studies and mechanistically informed interventional trials incorporating multimodal phenotyping, biomarkers of target engagement, and clinically relevant cardiovascular outcomes may help define the translational significance of neuroimmune mechanisms. A clearer understanding of when and where neuroimmune signaling becomes maladaptive may ultimately support the development of more precise strategies to promote inflammatory resolution while preserving protective immune responses.

## Glossary

ACh: acetylcholine
Ang II: angiotensin II
ATLO: adventitial tertiary lymphoid organ
AV: atrioventricular
β2-AR: β2-adrenergic receptor
CNS: central nervous system
CRACR2A: calcium release-activated calcium channel regulator 2A
CVDs: cardiovascular diseases
CXCL12: C-X-C motif chemokine ligand 12
HF: heart failure
HSPC: hematopoietic stem and progenitor cell
IL-1β: interleukin-1 beta
IL-1R: interleukin-1 receptor
IL-6: interleukin-6
IL-10: interleukin-10
IL-17: interleukin-17
Ly6C^hi^: lymphocyte antigen 6 complex, locus C high
M1: classically activated macrophage phenotype
M2: alternatively activated macrophage phenotype
MI: myocardial infarction
MRGPRX2: Mas-related G protein-coupled receptor X2
Mrgprb2: Mas-related G protein-coupled receptor B2
NE: norepinephrine
NETs: neutrophil extracellular traps
NLRP3: NOD-, LRR- and pyrin domain-containing protein 3
NPY: neuropeptide Y
NTS: nucleus of the solitary tract
PCO₂: partial pressure of carbon dioxide
PNS: parasympathetic nervous system
PO₂: partial pressure of oxygen
PVN: paraventricular nucleus
RVLM: rostral ventrolateral medulla
SNS: sympathetic nervous system
SPMs: specialized pro-resolving mediators
Th17: T helper 17 cell
TNF-α: tumor necrosis factor alpha
TNF-R: tumor necrosis factor receptor
TRPM2: transient receptor potential melastatin 2
α7nAChR: α7 nicotinic acetylcholine receptor
